# Platinum–nickel frame within metal-organic framework fabricated *in situ* for hydrogen enrichment and molecular sieving

**DOI:** 10.1038/ncomms9248

**Published:** 2015-09-22

**Authors:** Zhi Li, Rong Yu, Jinglu Huang, Yusheng Shi, Diyang Zhang, Xiaoyan Zhong, Dingsheng Wang, Yuen Wu, Yadong Li

**Affiliations:** 1 Department of Chemistry and Collaborative Innovation Center for Nanomaterial Science and Engineering, Tsinghua University, Beijing 100084, China; 2 Beijing National Center for Electron Microscopy, School of Materials Science and Engineering, Tsinghua University, Beijing 100084, China; 3 Center of Advanced Nanocatalysis (CAN-USTC), University of Science and Technology of China, Hefei, Anhui 230026, China

## Abstract

Developing catalysts that provide the effective activation of hydrogen and selective absorption of substrate on metal surface is crucial to simultaneously improve activity and selectivity of hydrogenation reaction. Here we present an unique *in situ* etching and coordination synthetic strategy for exploiting a functionalized metal-organic framework to incorporate the bimetallic platinum–nickel frames, thereby forming a frame within frame nanostructure. The as-grown metal-organic framework serves as a ‘breath shell' to enhance hydrogen enrichment and activation on platinum–nickel surface. More importantly, this framework structure with defined pores can provide the selective accessibility of molecules through its one-dimensional channels. In a mixture containing four olefins, the composite can selectively transport the substrates smaller than its pores to the platinum–nickel surface and catalyse their hydrogenation. This molecular sieve effect can be also applied to selectively produce imines, which are important intermediates in the reductive imination of nitroarene, by restraining further hydrogenation via cascade processes.

## Abstract (web summary)

Catalysts with improved hydrogen activation and substrate adsorption are required for advanced hydrogenation applications. Here, the authors fabricate metal organic framework coated platinum–nickel catalyst exhibiting enhanced hydrogenation activity and substrate selectivity due to molecular sieving effects.

## Graphical abstract

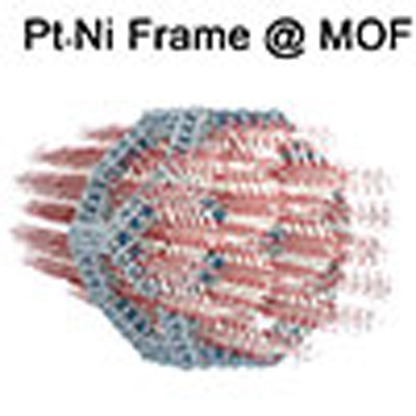

Hydrogenation reaction is a fundamental component in metal catalysis. How to improve the absorption and dissociation of H_2_ on metal surface is strictly related to the activity of hydrogenation reactions. Expanding the exposed metal sites as much as possible is an effective strategy to optimize the usage of precious metal, which is also a benefit for the activation of H_2_. For that sake, multitudinous structures such as hollow^1^,^2^, porous^3^, concave^4^,^5^ metallic structure with high surface area-to-volume ratio have been developed. Among them, frame-structured metal material has been demonstrated as one promising catalyst not only because all the reactive corners and edges can be maintained, but also the three-dimensional (3D) molecular accessibility which can facilitate the contact between H_2_ and metal^6^,^7^,^8^. Apart from the activity, promoting the selectivity of hydrogenation is another key concern for the design of nanocatalysts, which is mainly dependent on the absorption of substrate on metal surface. Owing to intensive research efforts focusing on surface science and catalysis, substantial factors such as crystal facet^9^, exposed defects^10^, interfaces^11^ and surface ligands^12^ have been discovered to influence the selective absorption of substrate on metal surface. Learning from the nature that carries out enzymatic transformations with excellent shape- and size selectivity, we believe metal-organic frameworks (MOFs) possessing tunable porosity and 3D nanoframe structure may impart molecular sieving to metal catalyst by controlling the diffusion of substrate, thereby tuning the selectivity of hydrogenation. Hence, the frame motif can be extended by coating a shell of MOF on the surface of metal frame to achieve unique frame within frame (frame @ frame) structure, which may endow new chances to achieve H_2_ enrichment and molecular sieving in metal catalysis simultaneously.

Dealloying process is a top-down strategy to carve the bimetallic structure at nanoscale. Driven by the different chemical reactivity of two metallic species, this versatile method has gained great success hitherto in constructing the bimetallic nanoframe^6^,^8^ structures. In contrast, the fabrication of MOFs is based on the cooperative assembly of organic linker and metal ions, which can be termed as bottom-up strategy. There is a severe drawback (often neglected) if dealloying strategy is adopted to construct bimetallic nanoframe. The carving process for generating interior vacancies and surface defects is strictly related to the dissolution of active metals. By whatever means necessary including oxidative etching which usually utilize oxidant^13^,^14^,^15^ or galvanic reaction involving the replacement between two different metals^16^,^17^, the active metals are consistently converted to ionic counterpart and abandoned in most cases^18^.

Herein, we take the advantages of both top-down and bottom-up strategies, using organic linkers to capture the abandoned Ni^2+^ ion during the dealloying process, to build a shell of MOFs on the surface of Pt–Ni alloy *in situ*. This unique frame @ frame nanostructure is expected to inherit the desirable properties of both Pt–Ni frames and MOFs.

## Results

### Synthesis and characterization

**Figure 1:**
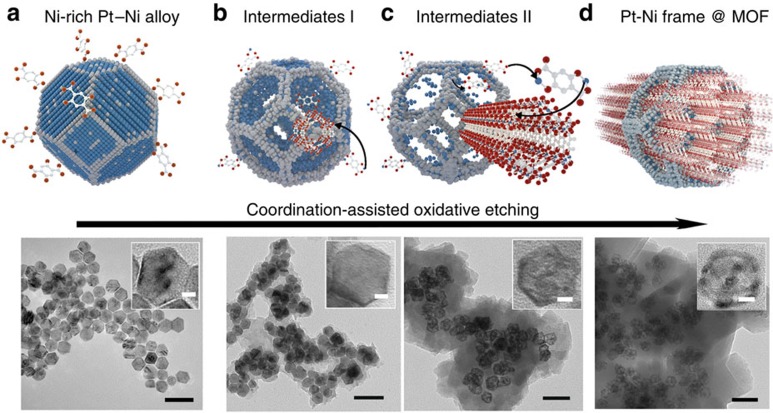
Scheme and corresponding TEM images of the coordination-assisted oxidative etching process. (**a**) Initial solid Pt–Ni polyhedra. (**b**) Pt–Ni frame @ MOF intermediates I. (**c**) Pt–Ni frame @ MOF intermediates II. (**d**) Final Pt–Ni frame @ MOF. The scale bars, 50 nm. (Insets are the magnified TEM images. The scale bars, 5 nm).

To produce this unique frame @ frame structure, we firstly prepared Ni-rich Pt–Ni alloy according to our previously reported method^19^. The starting Pt–Ni polyhedrons exhibit excellent monodispersity with average size of ∼20 nm and uniform truncated octahedral morphology (Fig. 1a). The polyvinyl pyrrolidone (PVP)-capped Ni-rich Pt–Ni nanoparticles (NPs) were submersed in dimethylformamide (DMF) to form a turbid solution, followed by adding a solution of 2,5-dioxidoterephthalate. During 12-h solvothermal process, three representative samples at 0.5, 4 and 12 h were collected and observed by transmission electron microscopy (TEM). In the first 0.5 h, a shell with lower contrast had emerged on the surface of Pt–Ni NPs (Fig. 1b). Successively, the initial truncated octahedral Pt–Ni alloy would evolve into hybrid structure that opens nanoframe located within a readily formed overlayer, while maintaining its original symmetry (Fig. 1c,d). That is, a well-defined MOF of Ni_2_dobdc (dobdc^4−^ (2,5-dioxidoterephthalate)), commonly known as Ni-MOF-74 (refs 20), was expected to be weaved on the surface of Pt–Ni nanoframes. The TEM image in low magnification demonstrated that the etched Pt–Ni frames could be incorporated fully within the matrices of *in situ*-formed MOF in a well-dispersed manner (Supplementary Fig. 1). The inherent process may relate to the following equations:


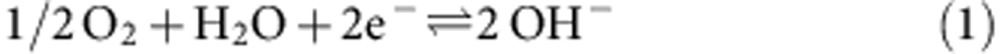



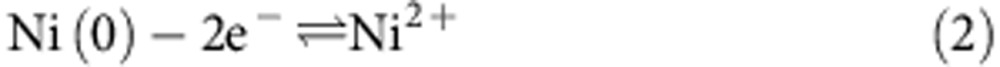



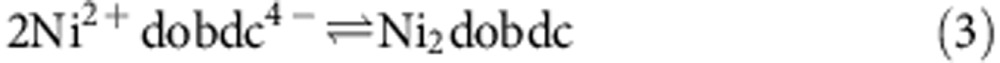


In detail, the oxidative etching of Pt–Ni alloy and the *in situ* nucleation of MOF-74 are two major interactive processes in this chemical etching, to some content maintaining synchronization. The two oxidation–reduction reactions shown in equations (1) and (2) are assigned to the electron transfer from Ni(0) to oxygen. Pt is a relatively inert element to oxygen compared with Ni, which determines the different diffusion rate during the etching process. The intrinsic formation mechanism of nanoframe may follow the Kirkendall effect^16^,^21^. In other words, the outward diffusion of Ni^2+^ and inward spread of voids dominate the whole etching process. Considering the unique Pt-segregated surface/Ni-segregated core structure reported^19^, the surface Pt shell cannot retain integrity as the etching of Ni proceeds, thereby generating the cavities inside the shell and driving the segregation of Pt at the frames. Apart from the electron transfer in oxidative etching of Ni to Ni^2+^, the species transfer of Ni from Pt–Ni nanoframe to Ni-MOF-74 is crucial to this frame @ frame structures. As schematically illustrated in Fig. 1, the Ni^2+^ on the surface of Pt–Ni nanoframe can be captured by the near-neighbour organic linker to form MOFs *in situ*. Once the precipitation of Ni-MOF-74 in equation (3) occurred, the equilibrium of oxidative etching reactions would be broken and shifted towards the generation of Ni^2+^ (equation (2)), thus largely accelerating the etching rate. To be accompanied by the Pt–Ni polyhedrons being etched to framework, another MOF would readily emerge and encapsulate the metallic framework within their matrices.

The composition evolution from Ni-rich Pt–Ni polyhedra to Pt-rich Pt–Ni frame and the nucleation of MOFs can be verified by X-ray diffraction (XRD) patterns, the inductively coupled plasma atomic emission spectroscopy (ICP-AES) and energy-dispersive X-ray spectra (Supplementary Table 1, Supplementary Figs 2 and 3). Compared with the initial Pt–Ni polyhedra, a set of peaks belonging to Ni-MOF-74 emerged after the structural evolution, indicating the successful coating of MOF on the etched Pt–Ni frame. The corresponding peaks derived from the organic groups of MOF overlayer, evidenced by the Fourier transform infrared spectra, perfectly match the signals of pure Ni-MOF-74 synthesized by previously reported method^22^ (Supplementary Fig. 4). After treating this composite with dilute acetic acid, the coating MOF can be removed by cutting off the coordination between phenolate and carboxylic oxygen atoms and Ni^2+^, thus leaving a bare Pt–Ni-framed structure (Supplementary Fig. 5). From the XRD patterns belonging to Pt–Ni frame, the face-centred cubic peaks for (111), (200) and (220) facets were found to shift towards lower 2*θ* values due to the increasing d spacing, which demonstrated the dissolution of Ni from parent Ni-rich Pt–Ni alloy. X-ray photoelectron spectroscopy (XPS) was used to trace the valence evolution in this coordination-assisted chemical etching (Supplementary Fig. 6). The Ni 2p and Pt 4f spectra of Pt–Ni polyhedra and Pt–Ni frame revealed that the surface Ni was partly oxidized to Ni^2+^ and most of the surface Pt retained metallic state. In the case of Pt–Ni frame @ MOF, the spectra for Ni showed the oxidation state was predominant, which was attributed to depletion of Ni(0) by coordination process. Meanwhile, the peaks assigned to metallic Pt were largely depressed, suggesting the seamless coating of MOFs will block the activated photoelectrons from coated Pt–Ni frame during the XPS measurements.

**Figure 2:**
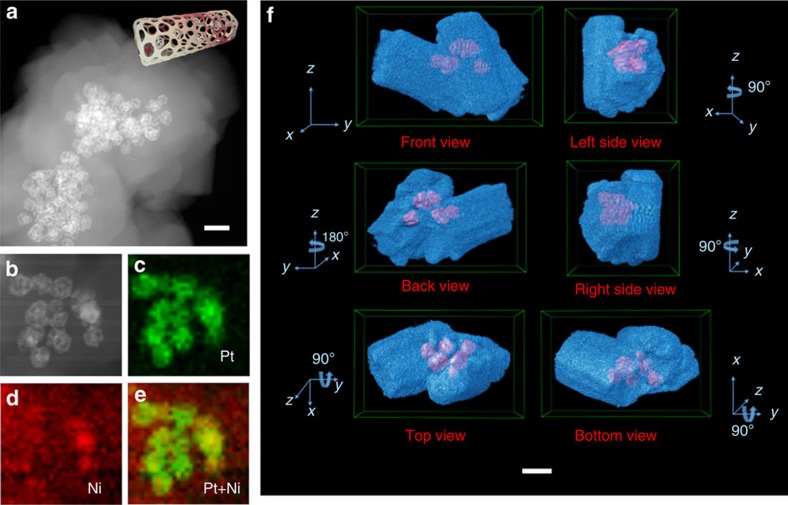
Characterization of Pt–Ni frame @ MOF. (**a**) HAADF-STEM image and ideal model of Pt–Ni frame @ MOF. The Pt–Ni frames were easily distinguished from the shrouding MOF matrix based on their different contrast. (**b**–**e**) Energy-dispersive X-ray elemental mapping results of Pt–Ni frame @ MOF, suggesting that Ni is homogeneously distributed throughout the entire nanostructure and Pt is concentrated where initial Pt–Ni alloy is situated. (**f**) Six projected images of three-dimensional visualization of tomographic reconstruction images of Pt–Ni frame @ MOF, demonstrating that the Pt–Ni frames were fully enshrouded by MOF-74. The scale bars, 50 nm.

The intrinsic morphology and spatial distribution of the frame @ frame structure were examined by the high-angle annular dark-field scanning transmission electron microscope (HAADF-STEM) for which they were well-suited because of the mitigatory electron radiation damage relative to bright field (Fig. 2)^23^,^24^. The unique features of encapsulated Pt–Ni frame including hollow interior and the interconnecting edges in space can be clearly seen in Fig. 2a. The frame structure was robust enough to resist the chemical etching process, lattice strain from the interface of Pt–Ni and MOF, and the post treatment such as centrifugation and drying. The corresponding elemental maps tell the Pt concentrated at where initial Ni-rich Pt–Ni alloy situated, whereas the Ni distribute homogeneously throughout the entire architecture (Fig. 2b–e). These results coincided to the line scan profiles (Supplementary Fig. 7) and reinforced that Ni could be preferentially etched from the Pt–Ni alloy versus Pt because of its more tendency towards oxidative etching in the presence of organic linkers. To provide more detailed information that describes the 3D imaging of real structure, tomographic data were reconstructed based on a series of 2D HAADF-STEM images, which were taken at consecutive tilt angles from −72° to 72° with each 4° tilt increment (Supplementary Fig. 8)^25^. The 3D tomography of frame @ frame structure (Supplementary Fig. 9) was achieved based on the differences in Z-contrast between Pt–Ni frame and MOF. Six projected images of 3D visualization of tomographic reconstruction of Pt–Ni frame @ MOF showed that the Pt–Ni frame were fully enshrouded by well-defined MOF-74 (Fig. 2f). More detailed animated voxel recording the rotation of tomogram is available in Supplementary Movie 1. Time-tracking scanning electron microscope images along with the structural evolution also support the seamless encapsulation of Pt–Ni NPs by grown MOFs (Supplementary Fig. 10). It is notable that this coordination-assisted chemical etching presented here can be readily generalized to octahedral Ni-rich Pt–Ni polyhedra (Supplementary Fig. 11) to construct another type of ‘concave Pt–Ni alloy @ frame' structure (Supplementary Fig. 12).

### Gas-sorption and catalytic properties

**Figure 3:**
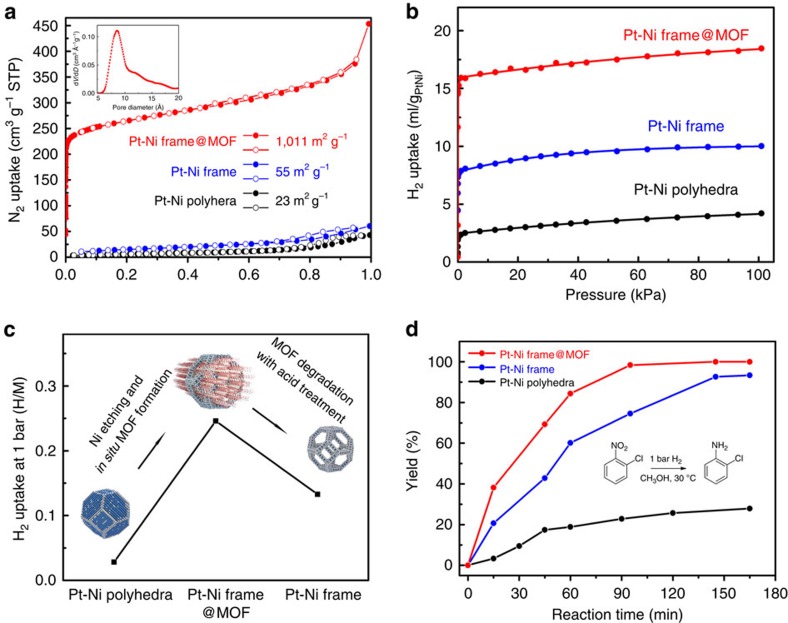
Gas-sorption properties and catalytic hydrogenation efficiencies. (**a**) Nitrogen-sorption isotherms at 77 K up to 1 bar. The filled and open symbols represent adsorption and desorption curves, respectively (**b**) H_2_ adsorption isotherms at 273 K up to 1 bar normalized by the mass of metal. Detailed interpretation of the calculation process is available in the ESI. (**c**) Comparison of H_2_ uptake at 273 K and 1 bar among three catalysts. (**d**) Yield (%) of 2-chloroaniline as a function of time in the selective hydrogenation of 1-chloro-2-nitrobenzene with Pt–Ni polyhedra, Pt–Ni frame and Pt–Ni frame @ MOF.

As is well-known, MOF-74, which owns characteristic honeycomb structure composed of 1D channel and open metal sites, is a widely used framework material due to its excellent chemical robustness and thermal stability^20^. Based on the profiles of thermogravimetric analysis (Supplementary Fig. 13), the linker decomposition starts at about 350 °C for Pt–Ni frame @ MOF composite, which is in good agreement with the as-prepared pure Ni-MOF-74. This grown MOF-74 not only enables evident confinement to strengthen the rigidity of Pt–Ni frames, but also behaves as a ‘breath shell' to largely enhance the uptake and enrichment of gas molecules. From the N_2_ adsorption and desorption isotherms on these catalysts, the frame @ frame structure exhibits 1 order of magnitude enhancement in Brunauer–Emmett–Teller surface area than the starting Pt–Ni polyhedra and bare Pt–Ni frame (Fig. 3a). According to the IUPAC definition, the sharp uptake at *P*/*P*_0_ from 10^−5^ to 10^−1^ indicates a standard type I isotherm with characteristic of 8.6-Å micropores and the additional uptake at high relative pressure of *P*/*P*_0_=0.9 implies the existence of macro pores generated by packing of frame @ frame nanostructures.

The H_2_ enrichment properties of frame @ frame structure were studied by H_2_ adsorption isotherms at 273 K, which were shown in Fig. 3b. By eliminating the amount of hydrogen absorption on pure Ni-MOF-74, the number of hydrogen atoms absorbed on each metallic atom (Pt(0)+Ni(0)) were normalized for Pt–Ni polyhedra, Pt–Ni frame and Pt–Ni frame @ MOFs separately (Supplementary Figs 14 and 15). An obvious increment from 0.03 H per metal atom absorbed in bare Pt–Ni polyhedra to 0.25 H per metal atom in Pt–Ni frame @ MOFs was observed after the encapsulation of MOF-74. In contrast, the removal of grown MOF-74 by acid treatment would result in a 46% degradation of H_2_ storage for the Pt–Ni frame. We found that this H_2_ enrichment can be applied to facilitate the catalytic efficiency of hydrogenation reaction. In our case of study, the selective hydrogenation of 1-chloro-2-nitrobenzene, which represents an important industrial conversion^26^,^27^, was selected to probe the structure-activity relationship of as-prepared frame @ frame catalysts. Since the diffusion problem for 1-chloro-2-nitrobenzene is expected to be negligible through the large pore apertures of MOF-74, the enhanced H_2_ storage does confer this frame @ frame catalyst even higher catalytic efficiency compared with the bare Pt–Ni frame. Actually according to the TEM and XRD measurements, this frame @ frame catalyst can retain its structural stability and catalytic activity after 10 runs of recycle measurement without significant decrease in 2-chloroaniline selectivity (Supplementary Figs 16–18).

## Discussion

**Figure 4:**
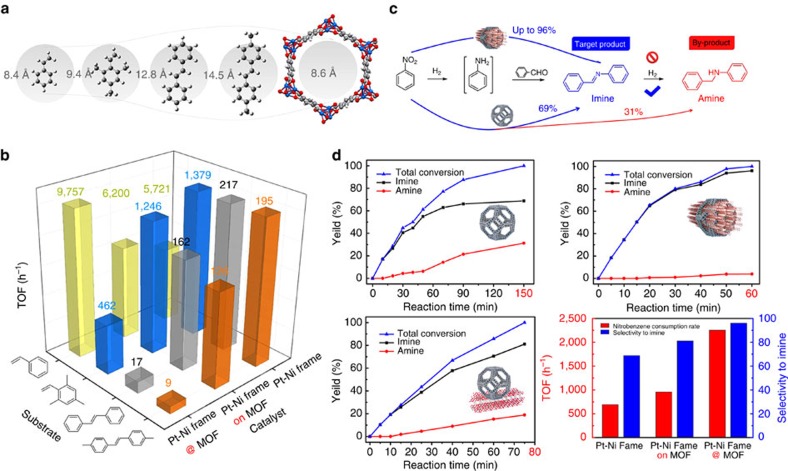
Size-selective catalytic behaviors of Pt–Ni frame @ MOF (**a**) Scheme showing the comparison of the maximum diameters of four representative substrates with the pore diameter of Ni-MOF-74. (**b**) Hydrogenation of styrene, 2,4,6-trimethylstyrene, *trans*-stilbene and 4,4′-dimethyl-*trans*-stilbene catalysed by three catalysts. (**c**) Scheme showing the size-selective catalysis in reductive imination of nitrobenzene. (**d**) Kinetic curves, TOFs and selectivity to imine in the cascade reductive imination of nitrobenzene catalysed by Pt–Ni frame, Pt–Ni frame on MOF and Pt–Ni frame @ MOF. The TOF values were calculated on the basis of the active sites measured from the CO chemsorption experiments.

Accordingly, this well-defined textural property may endow frame @ frame catalyst with molecular sieving to achieve efficient and selective hydrogenation for target products, if the size of substrate or product were ingeniously designed. Hydrogenation of a mixture containing styrene, 2,4,6-trimethylstyrene, *trans*-stilbene and 4,4′-dimethyl-*trans*-stilbene with different sizes (Fig. 4b) has been conducted to investigate the substrate-size selectivity in MOFs. As a comparison, catalysts composed of bare Pt–Ni nanoframe and Pt–Ni frame directly loaded on Ni-MOF-74 (Supplementary Fig. 19) were synthesized to further elucidate the effect of molecular sieving. Styrene molecules (8.4 Å) are small enough to diffuse through the pore apertures of MOF shells onto the Pt–Ni frame surface without hindrance. Therefore, it is reasonable that the frame @ frame structure can catalyse the hydrogenation of styrene with higher activity than Pt–Ni frame and Pt–Ni frame on MOF (Fig. 4b), resulting from the H_2_ enrichment. If the size of molecules increased, the diffusion of substrates would be strictly limited by the uniform pores of Ni-MOF-74. This confinement will significantly result in efficiency decay for Pt–Ni frame @ MOF catalyst by retarding the contact between olefins and Pt–Ni nanoframe inside. Apart from the deceased diffusion rates, the diffusion posture in channel could be limited, which was also detrimental to the hydrogenation of C=C bond in the middle if these olefins had to lay down in the channel^28^. In contrast, the hydrogenation of olefins catalysed by both of the Pt–Ni frame and Pt–Ni frame on MOF were barely affected because of the sufficient exposed Pt and Ni atoms outside.

Imines are important chemical intermediates due to their good electrophilicity for many important condensation, reduction and addition reactions^29^,^30^,^31^. The one-pot cascade reductive imination of nitroarenes with carbonyl compounds is highly attractive. Three main chemical transformations should be taken into consideration in this process: reduction of nitroarene to generate aniline, condensation of aniline and aldehyde and hydrogenation of the imine (Fig. 4c). Some substituted reductants such as carbon monoxide (CO) or CH_3_OH instead of H_2_ were usually introduced to prevent the over reductions of imines in previous reports^29^,^32^. Comparably, using hydrogen as a reductant to achieve one-pot reductive imination of nitroarene, which involves obvious size evolution from reactants to products, is more desirable and suitable for evaluating the performance of H_2_ enrichment and molecular sieving. As such, the catalytic behaviours of three catalysts with different topological structures (Pt–Ni frame, Pt–Ni frame on MOF and Pt–Ni frame @ MOF) were studied towards this cascade reaction. In the first step, the diffusion of nitroarene is unaffected for all of these catalysts because this small molecule can quickly diffuse onto the metal surface. Once aniline is formed through a reductive process, it will condense with aldehydes to provide target imines. In line with our conjecture, the frame @ frame structure is the most efficient catalyst, whose catalytic activity (based on surface metal atoms measured by CO titration, Supplementary Table 3) reaches 3.3 and 2.4 times higher than that of bare Pt–Ni nanoframe and Pt–Ni frame on MOF catalysts, respectively, to produce imines due to the superior H_2_ enrichment (Fig. 4d). The most important finding for this unique catalyst is that the over-reduction of imine, which produces N-phenylbenzylamine, can be effectively avoided. It is inferred that the uniform micropores derived from the grown MOFs can realize the size selectivity and suppress the diffusion of imine towards interior Pt–Ni frame once the condensation process is finished. Combining the outstanding H_2_ enrichment and molecular sieving derived from frame @ frame catalyst, imines could be efficiently and selectively produced in a cascade reaction by artfully modulating the reaction process. In addition, Ni nanocrystal and Ni-MOF-74 were tested to be catalytically inactive in the hydrogenation of 1-chloro-2-nitrobenzene, hydrogenation of styrene and reductive imination of nitrobenzene (Supplementary Table 2). The commercial Pt/C showed relatively higher hydrogenation efficiency (with same Pt loading) in the selective hydrogenation of 1-chloro-2-nitrobenzene. However, measuring the surface atoms by CO titration, the turnover frequencies (TOFs) towards hydrogenation of mixed olefins and reductive imination of nitroarenes are relatively lower for Pt/C. Further, the selectivity of commercial Pt/C to target imine product is much lower than that of Pt–Ni frame @ MOF, resulting from its non-restricted contact with substrate (Supplementary Fig. 20).

In conclusion, a novel frame @ frame structure can be sophisticatedly directed by combining the oxidative etching of Pt–Ni alloy and *in situ* precipitation of MOF together. The present design criteria enable this open structure efficient and multifunctional catalyst, namely, maximized the use of precious Pt at the active corners and edges of Pt–Ni bimetallic nanoframes, increased H_2_ enrichment which allows for more facile reactivity towards hydrogenation reaction, excellent molecular-size selectivity that originate from the grown microporous metal-organic frameworks. These findings based on the structural evolution of bimetallic nanostructure may also be applicable to many other bimetallic NPs @ MOFs catalysts, possibly offering a hint to simultaneously tune the activity, selectivity and durability.

## Methods

### Reagents

Analytical grade benzyl alcohol was obtained from Beijing Chemical Reagents, China. Pt(acac)_2_ (99%), Ni(acac)_2_ (99%), PVP (molecular weight (MW)=8,000), nitrobenzene, benzaldehyde, benzoic acid, styrene, 2,4,6-trimethylstyrene, *trans*-stilbene, biphenyl, anisole and Platinum (5% on carbon) were purchased from Alfa Aesar. 2,5-dihydroxyterephthalic acid, 4,4′-dimethyl-*trans*-stilbene and 1-chloro-2-nitrobenzene were acquired from TCI. Aniline was purchased from J.K Scientific. All of the chemicals used in this experiment were analytical grade and used without further purification.

### Characterizations

The crystalline structure and phase purity were determined using a Rigaku RU-200b X-ray powder diffractometer with CuKa radiation (*l*=1.5418 Å). The composition of the product was measured by the ICP-AES and energy-dispersive X-ray spectra. The catalysts' sizes and morphologies were analysed on a Hitachi H-800 TEM and a FEI Tecnai G2 F20 S-Twin high-resolution TEM. XPS experiments were performed on a ULVAC PHI Quantera microprobe. Binding energies (BE) were calibrated by setting the measured BE of C 1 s to 284.8 eV. H_2_ adsorption isotherms were measured using a Quantachrome Autosorb-1 volumetric instrument at 273 K. The temperature was maintained at 273 K during measurements by putting excess ice with water in Dewar flask. All sample was degassed over 8 h at 423 K under vacuum to remove adsorbed gas or moisture. Requisite amount of hydrogen was injected into the volumetric set-up at volumes required to achieve a targeted set of pressures^33^. N_2_ sorption isotherms were performed in a Quantachrome Autosorb-1 at 77 K up to 1 bar. Before measurement, all samples were degassed over 8 h at 423 K under vacuum. Brunauer–Emmett–Teller surface area were obtained by analysing nitrogen adsorption isotherm. Pore size distributions were determined from the adsorption data based on the Horvath–Kwazoe model for cylinder pore geometry. Fourier transform infrared spectra were recorded on a Bruker-VERTEX 70 spectrometer. Thermogravimetry analyses were performed on Netzsch STA 449F3 thermogravimetric analyser over a temperature range of 40–850 °C at a heating rate of 10 °C min^−1^ in nitrogen atmosphere. Scanning electron microscopy was performed with a Hitachi SU-8010 instrument. A FEI Titan 80–300 TEM equipped with a spherical aberration (Cs) corrector for the objective lens working at 300 kV was used for collecting the HAADF-STEM tomography tilt series, which consisted of 37 HAADF-STEM images at the tilt range from −72° to 72° at a tilt increment of 4°. Simultaneous iterative reconstruction technique in FEI Inspect3D software was used for 3D reconstruction. Chimera software was employed to generate the 3D volume rendering of the reconstructions and analysis of the volumes.

### Preparation of truncated octahedral Pt–Ni alloy and octahedral Pt–Ni alloy

In a typical synthesis of Pt–Ni truncated octahedral nanocrystals, Pt(acac)_2_ (40 mg), PVP (MW=8,000) (400 mg), Ni(acac)_2_ (250 mg) and aniline (0.5 ml) were dissolved in 25 ml of benzyl alcohol, followed by 10 min of vigorous stirring. The resulting homogeneous green solution was transferred into a 50-ml Teflon-lined stainless-steel autoclave. The sealed vessel was then heated at 180 °C for 12 h before it was cooled down to room temperature. The products were separated via centrifugation and further purified by an ethanol–acetone mixture. In a typical synthesis of Pt–Ni octahedral nanocrystals, Pt(acac)_2_, (40 mg), PVP (MW=8,000, 400 mg), Ni(acac)_2_ (250 mg) and benzoic acid (250 mg) were dissolved in 25 ml of benzyl alcohol, followed by 10 min of vigorous stirring. The resulting homogeneous green solution was transferred into a 50-ml Teflon-lined stainless-steel autoclave. The sealed vessel was then heated at 180 °C for 12 h before it was cooled down to room temperature. The products were separated via centrifugation and further purified by an ethanol–acetone mixture.

### Preparation of *in situ*-grown Pt–Ni frame @ Ni-MOF-74

In a typical synthesis of Pt–Ni frame @ Ni-MOF-74, as-prepared truncated octahedron-shaped Pt–Ni nanoalloys (containing 1 mg Pt; based on inductively coupled plasma mass spectrometry (ICP-MS) measurement)) were dispersed in 1 ml DMF, and an appropriate amount of dihydroxyterephthalic acid (35 mg in 7.5 ml DMF) was added. The resulting solution was transferred into a 10-ml Telfon-lined stainless-steel autoclave. After stirring for 10 min, the sealed vessel was heated at 110 °C for 12 h before it was cooled down to room temperature. The *in situ*-grown Pt–Ni frame @ Ni-MOF-74 (Pt–Ni frame @ MOF) was obtained after washing and centrifugation by deionized water and methanol for several times. The obtained Pt–Ni frame @ Ni-MOF-74 was kept immersed in methanol for 5 days; the solvent was changed for fresh methanol once a day. Finally, the Pt–Ni frame @ Ni-MOF-74 was heated under vacuum at 150 °C and stored in a dry box for further use. In a typical synthesis of concave Pt–Ni @ Ni-MOF-74, the as-prepared octahedron-shaped Pt–Ni nanoalloys (containing 1 mg Pt; based on ICP-MS measurement) were dispersed in 0.5 ml DMF, and 7 ml DMF containing DOT (55 mg) and PVP (MW=30,000, 80 mg) was added. The resulting solution was transferred into a 10-ml Telfon-lined stainless-steel autoclave. After stirring for 10 min, the sealed vessel was then heated at 100 °C for 12 h before it was cooled down to room temperature. The *in situ*-grown concave Pt–Ni @ Ni-MOF-74 was obtained after washing and centrifugation by deionized water and methanol for several times. The obtained concave concave Pt–Ni @ Ni-MOF-74 was kept immersed in methanol for 5 days; the solvent was changed for fresh methanol once a day. Finally, the concave Pt–Ni @ Ni-MOF-74 was heated under vacuum at 150 °C and stored in a dry box for further use.

### Preparation of bare Pt–Ni frame

In a typical procedure, Pt–Ni frame @ MOF (containing 1 mg Pt; based on ICP-MS measurement)) was dispersed in 10 ml H_2_O. Into this solution, 10 ml dilute acetic acid (50%) was added. The resulting solution was stirred vigorously for 8 h in 30 °C to achieve complete decomposition of Ni-MOF-74. The Pt–Ni frames were obtained after washing and centrifugation by deionized water and methanol.

### Preparation of Ni-MOF-74 and Pt–Ni frame on Ni-MOF-74

Pure Ni-MOF-74 was synthesized by a modified condition from the literature procedures^34^. To a solution of 2,5-dihydroxyterephthalic acid (148 mg, 0.75 mmol) in THF (2.5 ml), a solution of nickel(II) acetate tetrahydrate (375 mg, 1.5 mmol) in water (25 ml) was added. The suspension was stirred and ultrasonicated until homogenous. The resulting solution was transferred into a 10-ml Telfon-lined stainless-steel autoclave. The sealed vessel was then heated at 110 °C in a preheated oven for 72 h before it was cooled down to room temperature. Ni-MOF-74 was obtained after washing and centrifugation with deionized water and methanol. In a typical preparation of Pt–Ni frame on Ni-MOF-74 (Pt–Ni frame on MOF), bare Pt–Ni frames were dispersed in methanol to form a suspension with a concentration of 5 mg Pt per 10 ml methanol. Into this solution, 20 mg Ni-MOF-74 was added and stirred at room temperature for 8 h. The composites were then separated via centrifugation and washed with methanol for several times. The obtained Pt–Ni frame on MOF was kept immersed in methanol for 5 days; the solvent was changed for fresh methanol once a day. Finally, the Pt–Ni frame @ Ni-MOF-74 was heated under vacuum at 150 °C and stored in a dry box for further use.

### Typical procedure for the catalytic hydrogenation of 1-chloro-2-nitrobenzene

About 35 μl 1-chloro-2-nitrobenzene (0.3 mmol) in 1.5 ml methanol and the catalyst (contain 0.005 mmol Pt, 1.6 mol %) were added in a 10-ml round flask. The round flask was purged with H_2_ to completely remove air from the reactor. Then, the reaction mixture was stirred at 30 °C under 1 bar H_2_. The progress of the reaction was monitored by gas chromatography (GC)-MS and the extent of conversion was determined on the basis of the ratio of area of substrate and product by an external standard method.

### Typical procedure for the catalytic hydrogenation of olefins

Hydrogenation of olefins was carried out in THF solution under 1 bar H_2_. In a typical procedure, the catalysts containing 0.0025, mmol Pt was loaded into a 10-ml round flask and THF (1.5 ml) was added to the reactor. The mixture was sonicated homogenously before mixed olefins (styrene, 2,4,6-trimethylstyrene, *trans*-stilbene and 4,4′-dimethyl-*trans*-stilbene) (0.1 mmol for each component) were introduced. Afterward, 0.1 mmol of biphenyl was also added as internal standard. The round flask was purged with H_2_ to completely remove air from the reactor and the reaction was allowed to proceed at 30 °C under 1 bar H_2_. The progress of the reaction was monitored by GC-MS. Hydrogenation rates for olefins were calculated on the basis of the consumption rates for the substrates. The TOFs were calculated using the following equation:


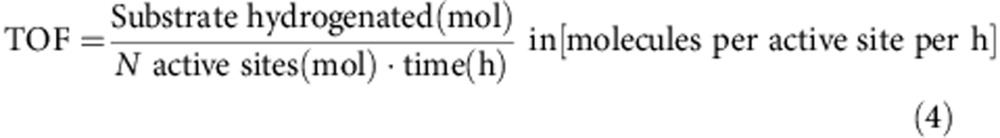


The active sites of the catalysts were measured by CO titration experiments.

### Typical procedure for the cascade reductive imination of nitroarenes

First, 22 μl nitrobenzene (0.2 mmol) and 35 μl benzaldehyde (0.3 mmol) in 2 ml ethanol and the catalyst (contain 0.005 mmol Pt, 2.5 mol %) were added in a 10-ml round flask. The round flask was purged with H_2_ to completely remove air from the reactor. Then, the reaction mixture was stirred at 30 °C under 1 bar H_2_. The progress of the reaction was monitored by GC-MS^29^,^35^ using anisole as an internal standard. Reaction rates for cascade reductive imination of nitroarenes were calculated on the basis of the consumption rates for the nitrobenzene. Conversion was defined as the mole ratio of converted nitrobenzene to starting nitrobenzene. The selectivity of imines based on nitrobenzene was calculated according to equation (5).


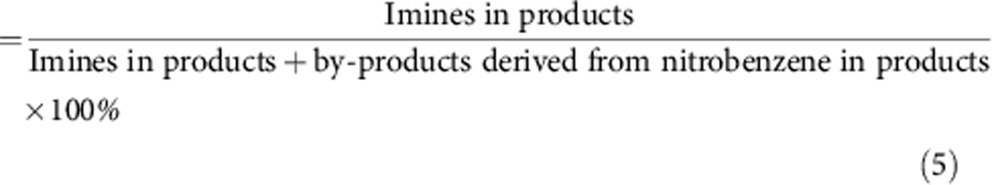


### Calculated maximum diameters of selected molecules

For olefins considered in this research, theoretical computations were performed with a Gaussian 03W programme package using density functional theory. Geometry optimizations and harmonic vibrational frequencies are computed with the B3LYP functional. Molecular lengths were measured as the distance between the two farthest apart atoms plus an estimate of the van der Waals radii of hydrogen (1.2 Å)^36^,^37^.

### CO titration experiments

The numbers of active sites on the surface of catalysts were determined from CO titration using a catalyst analyser (BEL-A, Japan) with a mass spectrometer (Inprocess Instruments, GAM200) as detector at 323 K. Prior to CO titration, the catalysts (containing *ca.* 2.5–3 mg metal) were treated at 423 K for 60 min and then cooled to 323 K under a argon flow (40 ml min^−1^). The CO uptake was measured by the decrease in the peak areas induced by chemsorption compared with the area of a calibrated volume. The metal dispersion was calculated assuming a stoichiometry of one CO molecule per surface metal atom (metal atom=Pt, Ni).

## Additional information

**How to cite this article:** Li, Z. *et al*. Platinum–nickel frame within metal-organic framework fabricated *in situ* for hydrogen enrichment and molecular sieving. *Nat. Commun.* 6:8248 doi: 10.1038/ncomms9248 (2015).

## Supplementary Material

Supplementary Figures, Supplementary Tables and Supplementary References Supplementary Figures 1-20, Supplementary Tables 1-3 and Supplementary References

Supplementary Movie 1 Rotation of the 3D Pt-Ni frame @ MOF tomography.
