# Supplementary material for: Platinum–nickel frame within metal-organic framework fabricated in situ for hydrogen enrichment and molecular sieving
Source: Nat Commun. 2015 Sep 22;6:8248. doi: 10.1038/ncomms9248 (PMC4595627; doi:10.1038/ncomms9248)
Supplement: Supplementary Figures, Supplementary Tables and Supplementary References — Supplementary Figures 1-20, Supplementary Tables 1-3 and Supplementary References [file ncomms9248-s1.pdf]

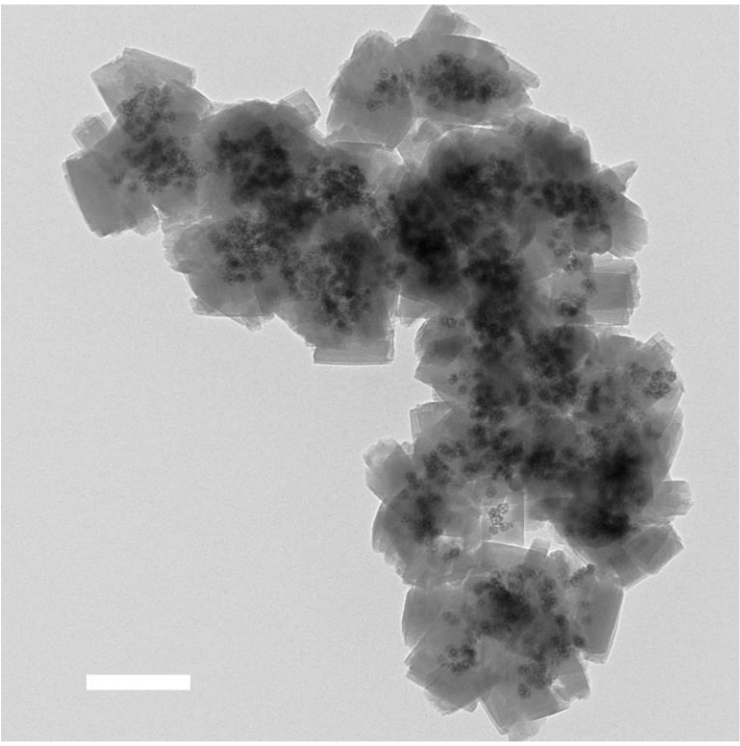

Supplementary Figure 1. Low-magnification TEM image of Pt-Ni frame @ MOF. The scale bar is 200 nm.

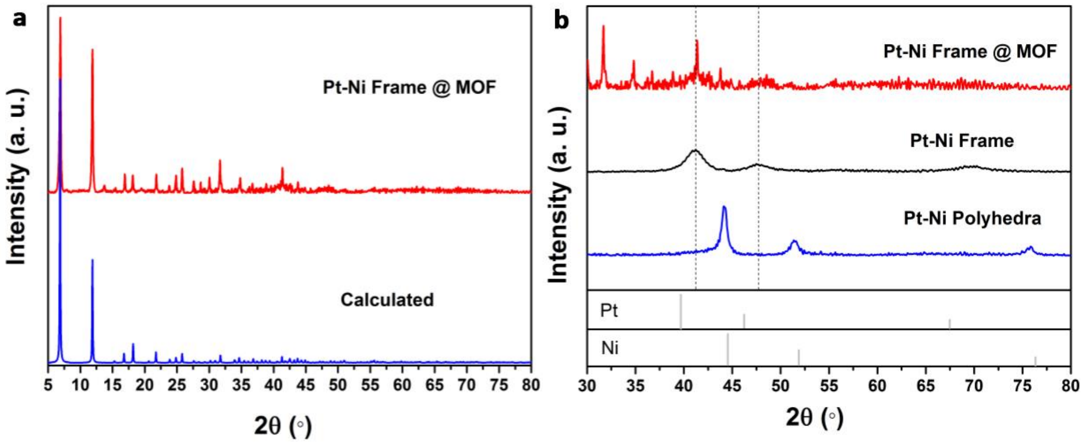

Supplementary Figure 2. (**a**) XRD patterns of the Pt-Ni @ MOF and the simulated Ni-MOF-74 pattern. (**b**) XRD patterns of Pt-Ni @ MOF, Pt-Ni frames and Pt-Ni polyhedra.

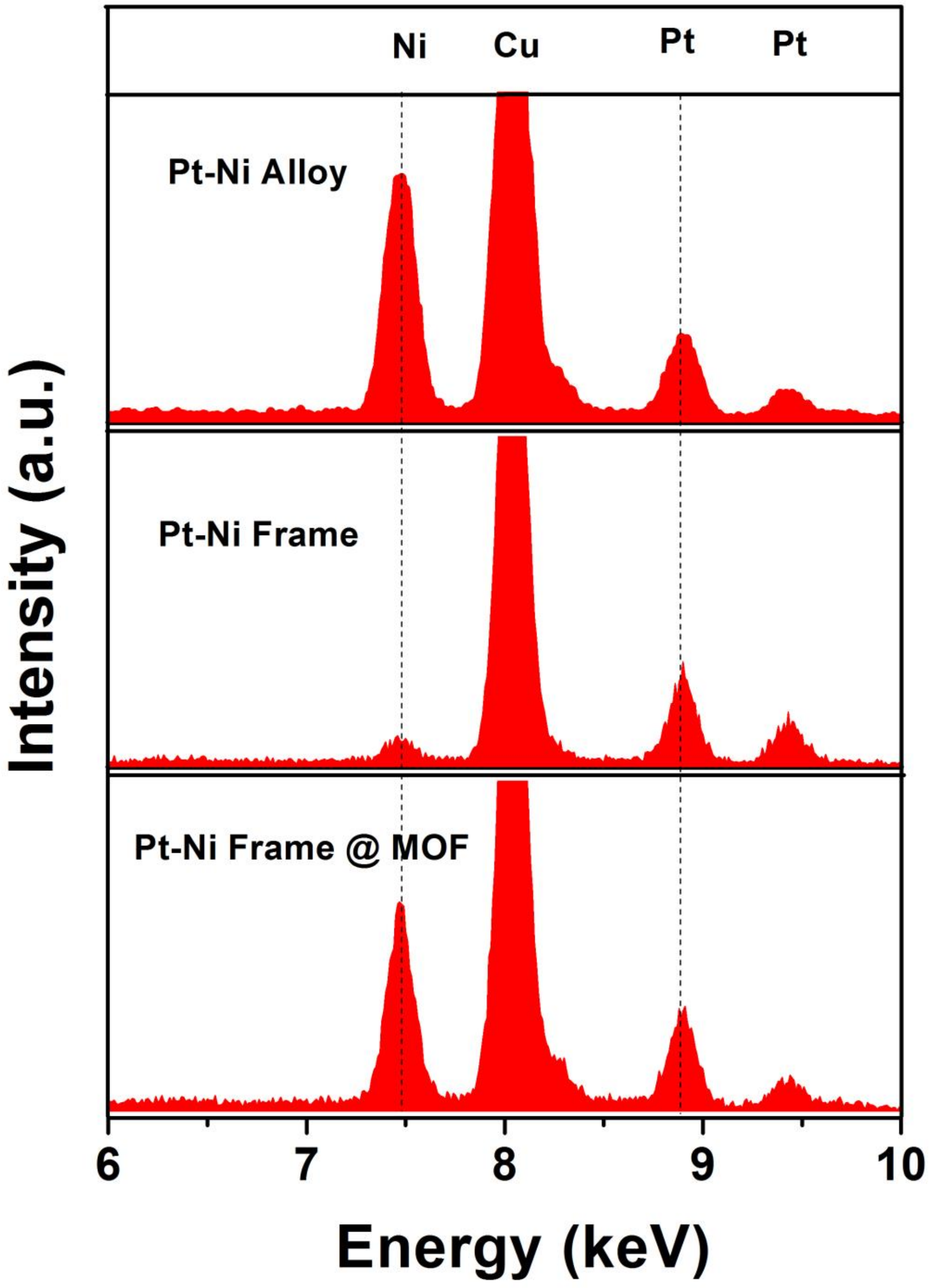

Supplementary Figure 3. EDX spectra of Pt-Ni frame @ MOF, Pt-Ni frame and Pt-Ni polyhedra.

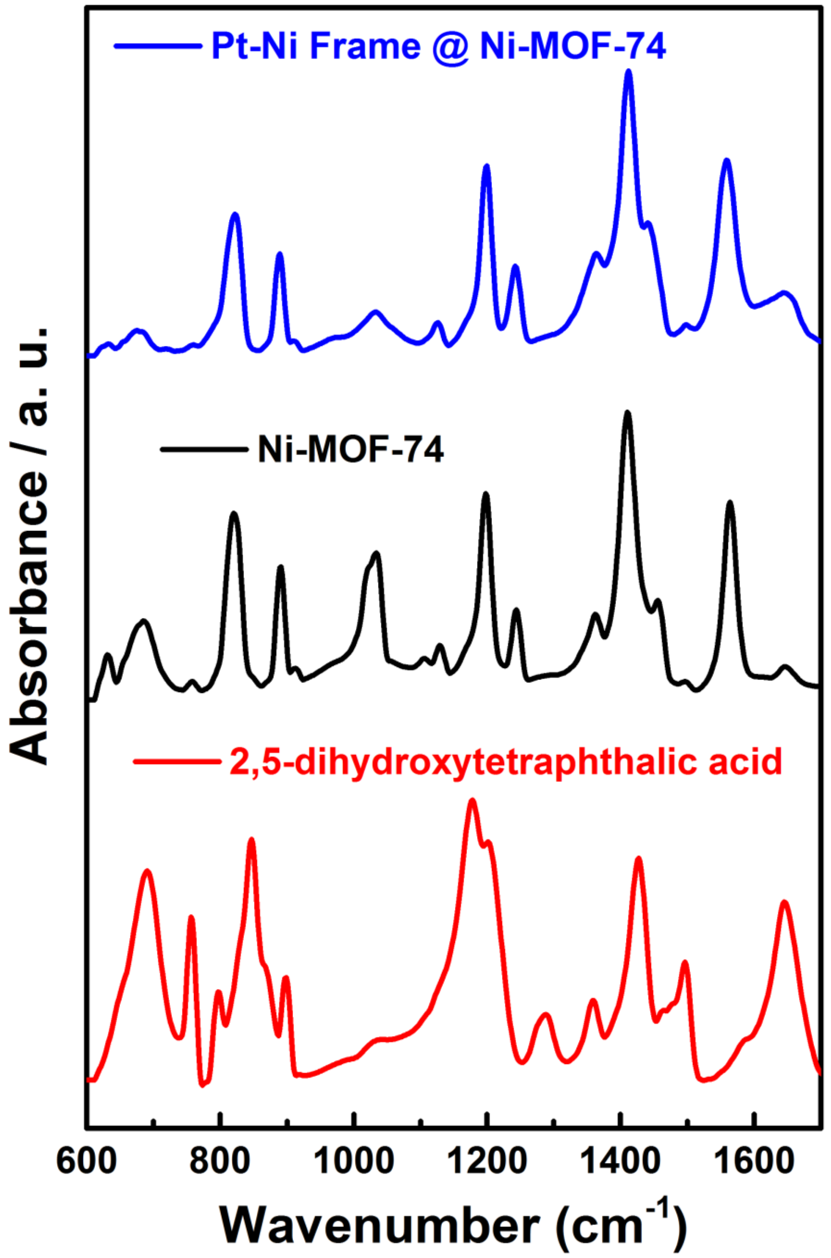

Supplementary Figure 4. The fingerprint region of the IR spectra registered for the Pt-Ni frame @ MOF, pure Ni-MOF-74 and the linker 2,5-dihydroxyterephthalic acid. IR spectra of the linker and pure Ni-MOF-74 are remarkably different while there shows a good agreement in band position and relative intensity between Pt-Ni frame @ MOF and pure Ni-MOF-74.

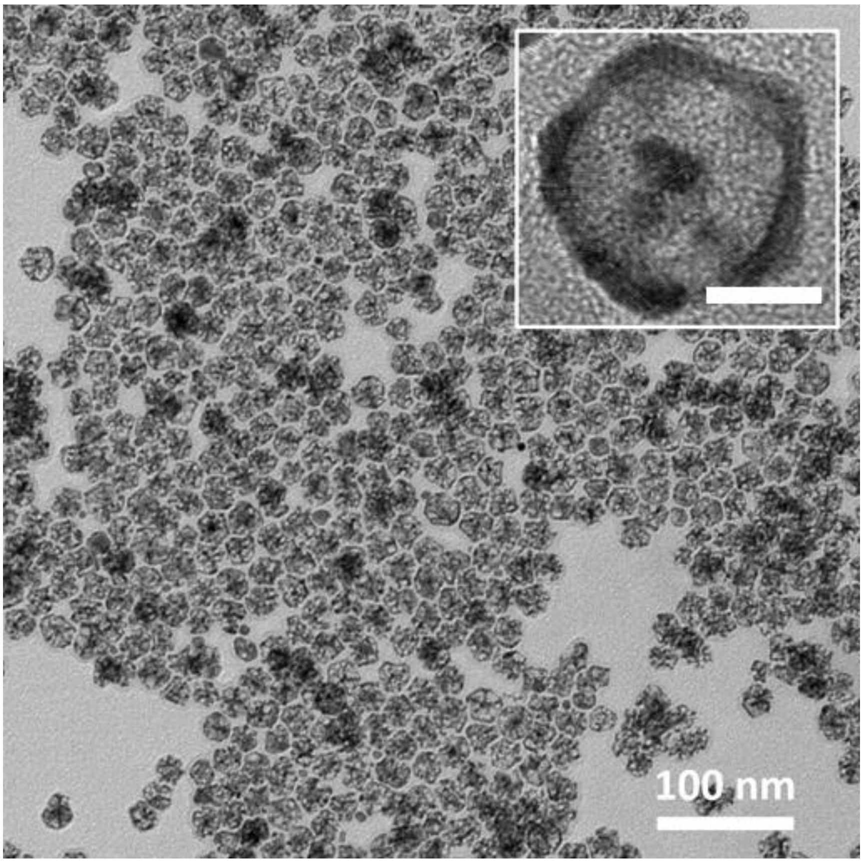

Supplementary Figure 5. TEM image of bare Pt-Ni frame and the scale bar is 100 nm. Inset is the magnified TEM image. The scale bar is 10 nm.

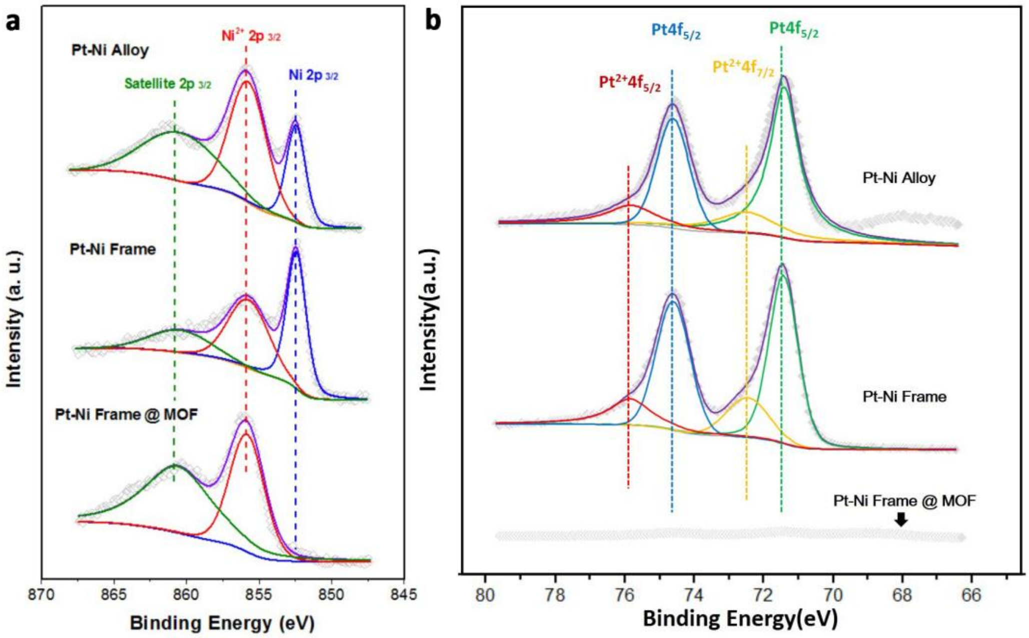

Supplementary Figure 6. (**a**) Ni 2p3/2 and (**b**) Pt 4f XPS spectra of Pt-Ni polyhedra, Pt-Ni frame, and Pt-Ni frame @ MOF.

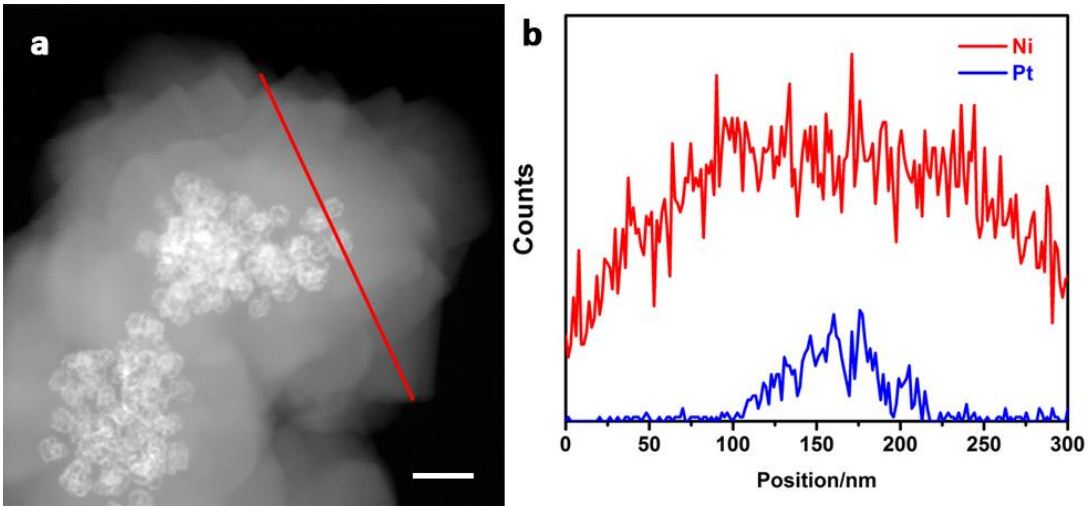

Supplementary Figure 7. (**a**) HAADF-STEM image and (**b**) cross-sectional compositional line profiles of Pt-Ni frame @ MOF. The scale bar is 10 nm.

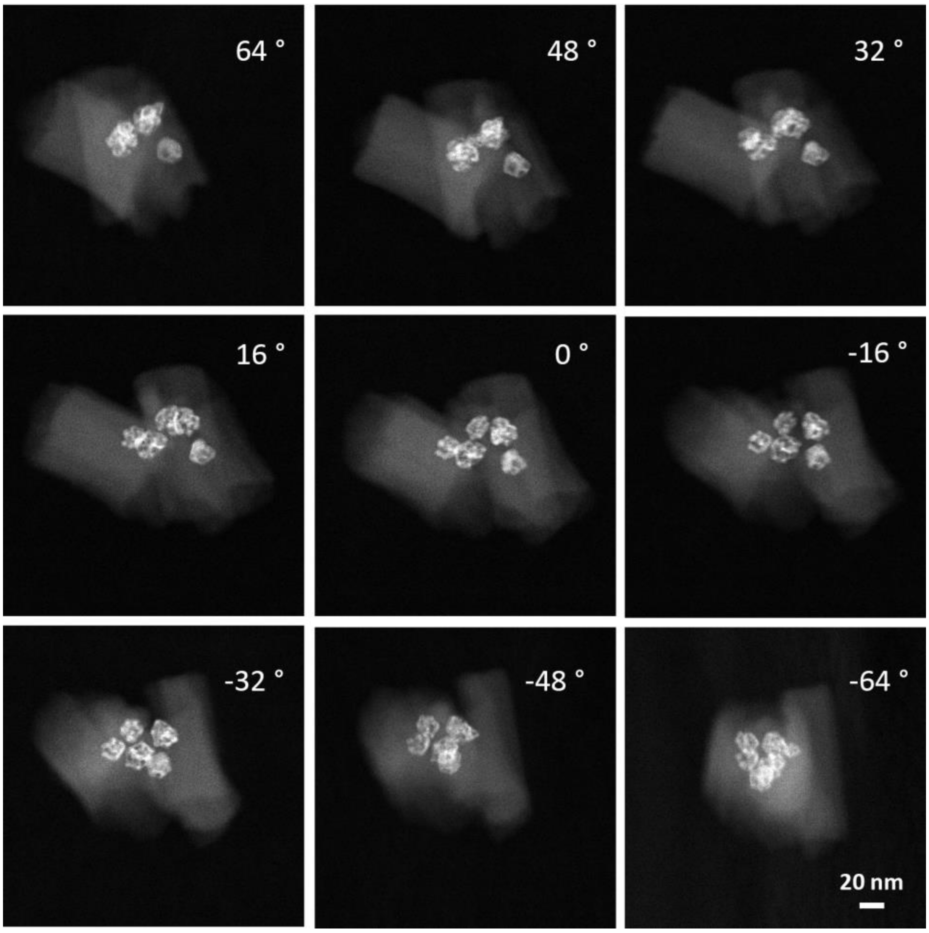

Supplementary Figure 8. HAADF STEM images of Pt-Ni frame @ MOF taken at representative tilt angles.

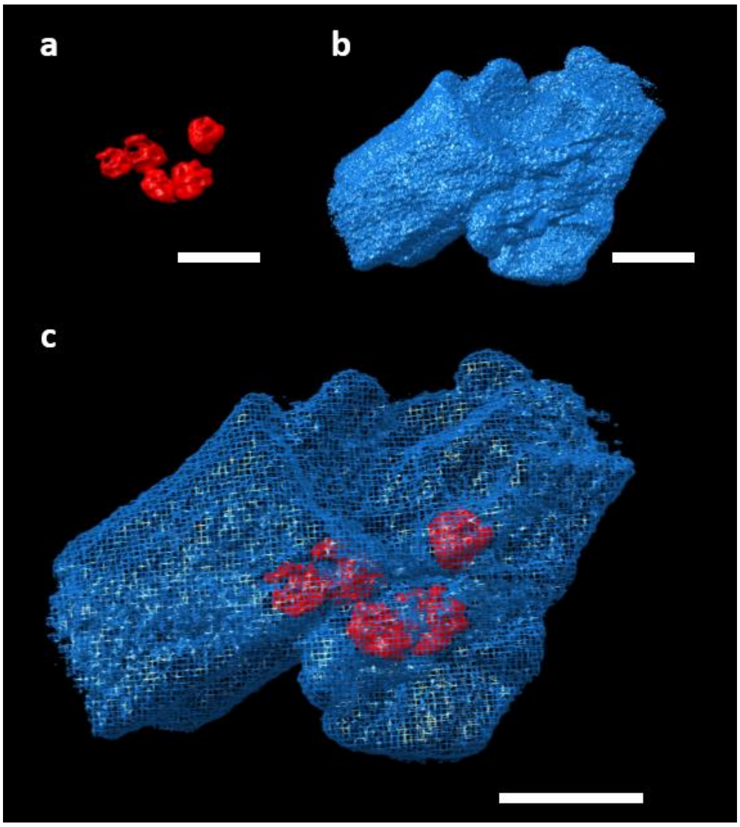

Supplementary Figure 9. Tomographic reconstruction of Pt-Ni frame @ MOF. The scale bars are 50 nm.

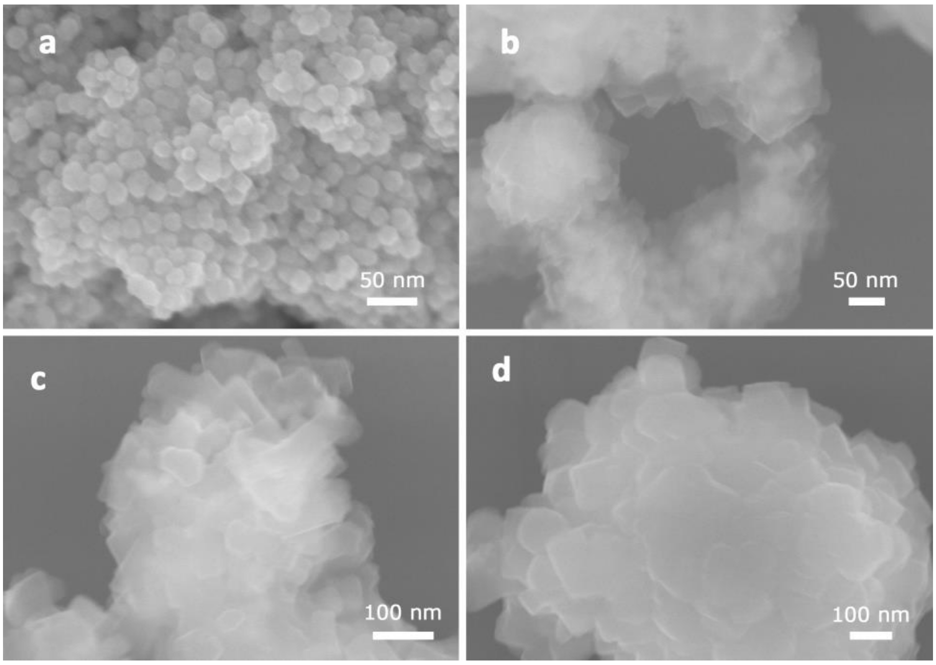

Supplementary Figure 10. SEM images showing morphology evolution from Pt-Ni polyhedra to Pt-Ni frame @ MOF sampled at different reaction stages: (**a**) 0 (**b**) 2 (**c**) 4 (**d**) 12 h.

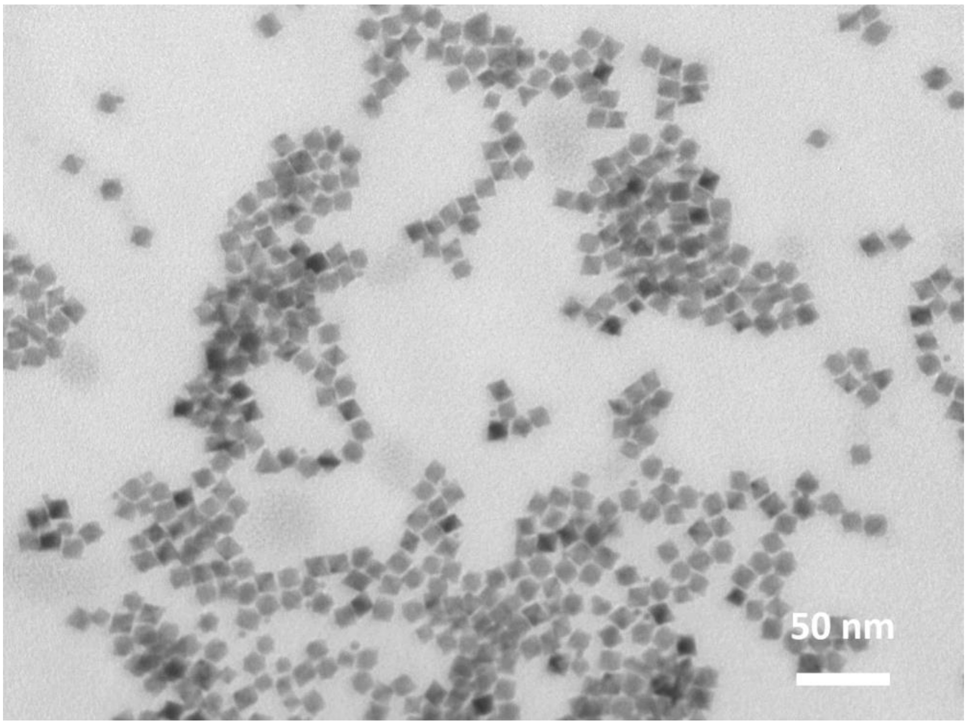

Supplementary Figure 11. TEM image of as-obtained octahedral Pt-Ni polyhedra.

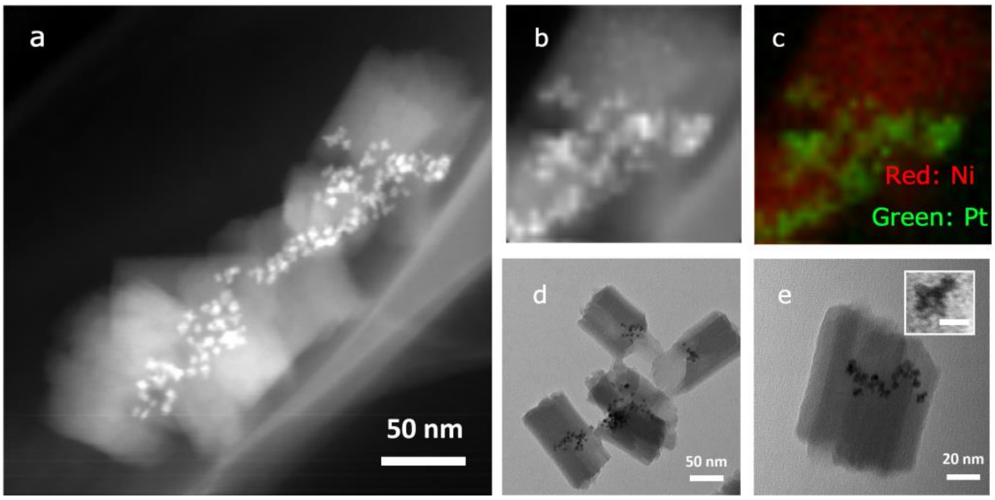

Supplementary Figure 12. (**a**), (**b**)HAADF-STEM image, (**c**)EDS mapping image and (**d**), (**e**)TEM image of concave Pt-Ni alloy @ MOF. Inset is the magnified TEM image showing the concave morphology of Pt-Ni alloy. The scale bar is 5 nm.

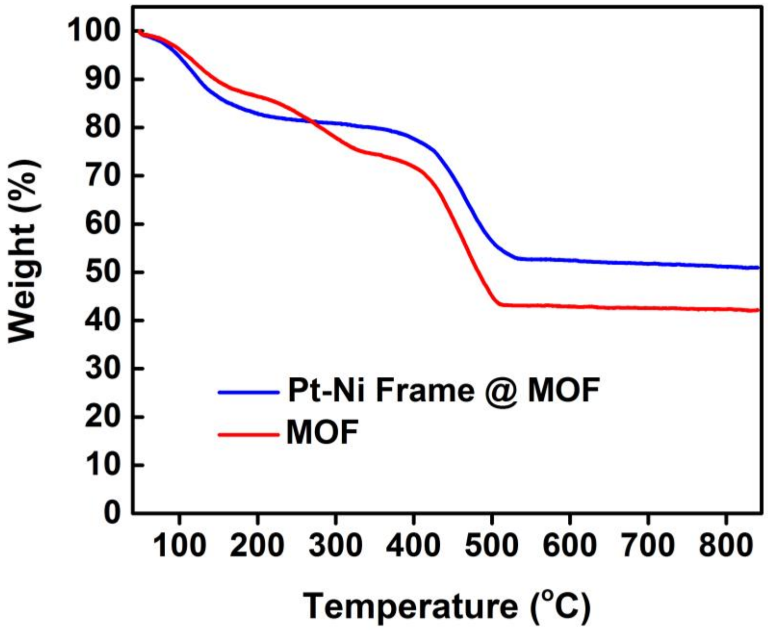

Supplementary Figure 13. TGA of Pt-Ni Frame @ MOF and Ni-MOF-74. Data were collected under flowing nitrogen using a temperature ramp of 10℃ • min^{-1}.

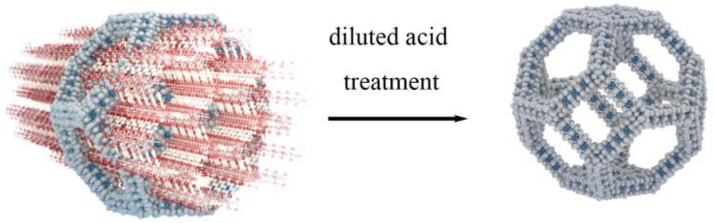

Supplementary Figure 14. Scheme showing the structural transformation from Pt-Ni frame @ MOF to bare Pt-Ni frame by acid treatment. From ICP-MS measurement, the Pt:Ni molar ratios within Pt-Ni frame @ MOF and bare Pt-Ni nanoframe are 1:6.7 and 1:0.5, repectively. Hence, the molar ratio of Pt : Ni (in nanoframe) : Ni(in MOF) is 1 : 0.5 : 6.2. As the stoichiometric ratio of Ni : ligand in Ni-MOF-74 is 2:1, the molecular formula of Pt-Ni frame @ MOF should be $PtNi_{0.5}(Ni_2L)_{3.1}$ and the mass fractions of Pt-Ni frame (core) and MOF (shell) could be obtained according to equation (1) and (2).

$$\omega_{PtNi} = \frac{(195.1+58.7*0.5)}{(195.1+58.7*0.5)+(58.7*2+198.1)*3.1} = 0.19 \quad (1)$$

$$\omega_{MOF} = \frac{(58.7*2+198.1)*3.1}{(195.1+58.7*0.5)+(58.7*2+198.1)*3.1} = 0.81 \quad (2)$$

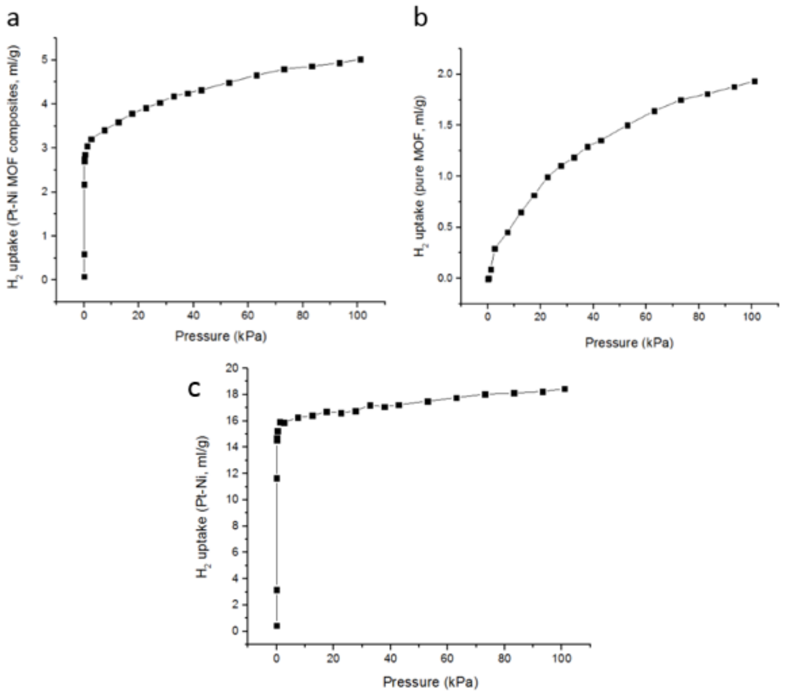

Supplementary Figure 15. Hydrogen adsorption capacity of (**a**) Pt-Ni frame @ MOF and (**b**) pure MOF. Calculated hydrogen adsorption capacity (**c**) of Pt-Ni frame in the Pt-Ni frame @ MOF. For the Pt-Ni frame @ MOF composites ($C_{PtNi\ core\text{-}MOF}$), the uptake capacity of H_2 were suggested to contain two parts: $C_{PtNi\ core}$ ($mL\cdot g^{-1}$) for the encapsulated Pt-Ni alloy core and C_{MOF} ($mL\cdot g^{-1}$) for MOF. The total uptake $C_{PtNi\ core\text{-}MOF}$ ($mL\cdot g^{-1}$) was considered to be the weighted arithmetic mean of $C_{PtNi\ core}$ and C_{MOF}. Hence, the calculated $C_{PtNi\ core}$ can be obtained by equation (3).

$$C_{PtNi\ core} = (C_{PtNi\ core\text{-}MOF} - C_{MOF} \cdot \boldsymbol{\omega}_{MOF}) / \boldsymbol{\omega}_{PtNi\ core} \qquad (3)$$

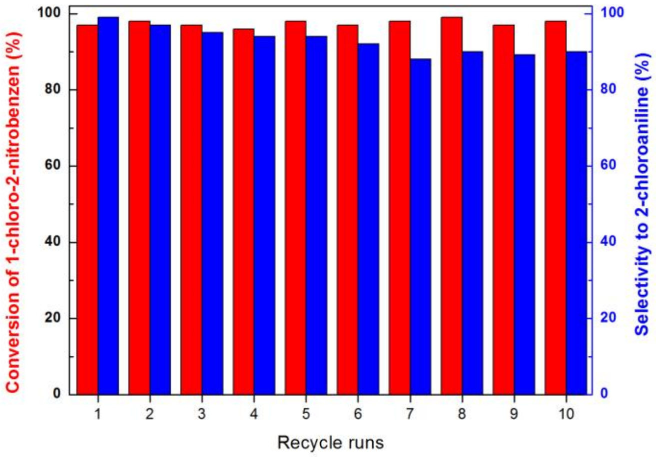

Supplementary Figure 16. The recycle experiment of selective hydrogenation of 1-chloro-2-nitrobenzene. Reaction conditions: 1-chloro-2-nitrobenzene (0.3 mmol), [Pt] (1mg, 1.6 mol%), CH_3OH (1.5 mL), PH_2: 1 bar, T: 30 ℃, 90min.

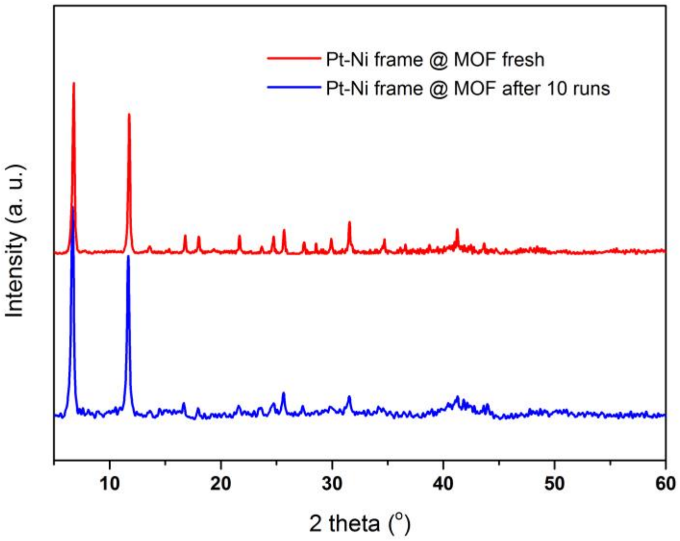

h

Supplementary Figure 17. PXRD of Pt-Ni frame @ MOF after 10 catalytic runs.

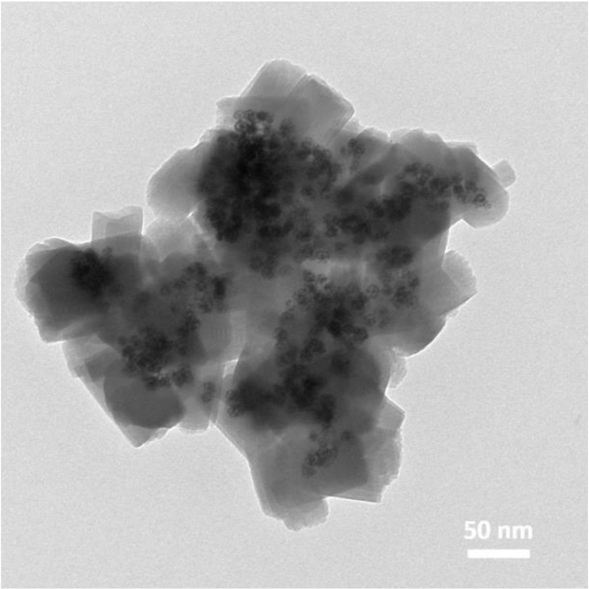

Supplementary Figure 18. TEM image of Pt-Ni frame @ MOF after 10 catalytic runs.

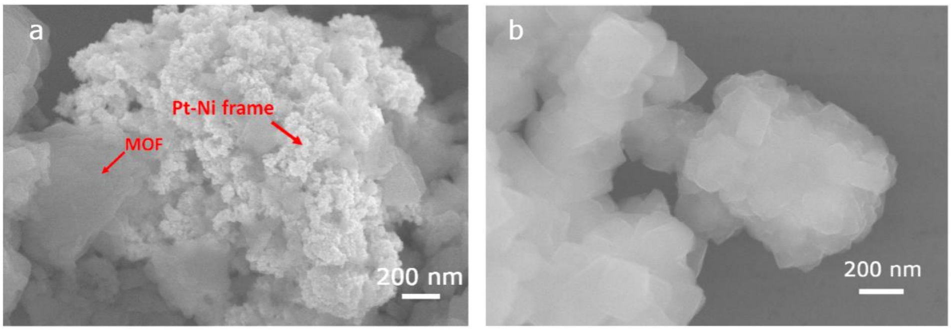

Supplementary Figure 19. SEM images of (a) the Pt-Ni frame on MOF and (b) Pt-Ni frame @ MOF catalysts, showing the different topological structure.

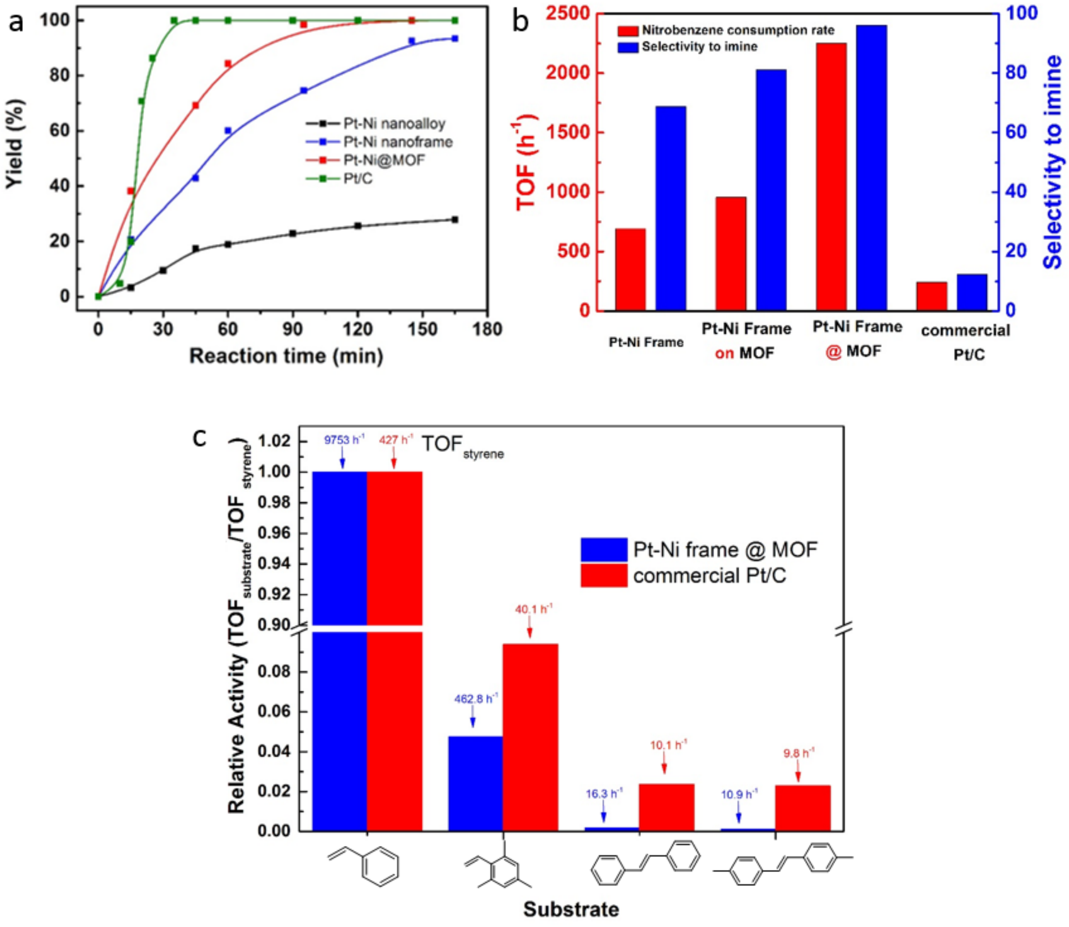

Supplementary Figure 20. The catalytic activity of commercial Pt/C compared with Pt-Ni alloy and Pt-Ni frame @ MOF in the hydrogenation of 1-chloro-2-nitrobenzene, reductive imination of nitrobenzene, and hydrogenation of olefins. (**a**) 1-chloro-2-nitrobenzene (0.3 mmol), catalysts [Pt] (0.005 mmol), CH_3OH (1.5 mL), PH_2: 1 bar, T: 30 ℃. (**b**) nitrobenzene (0.2 mmol), benzaldehyde (0.3 mmol), catalysts containing [Pt] (0.005 mmol), C_2H_5OH (2 mL), PH_2: 1 bar, T: 30 ℃. The TOF values were calculated based on the active sites measured from the CO titration experiments. (**c**) olefins (styrene, 2,4,6-trimethylstyrene, trans-stilbene, 4,4'-dimethyl-trans-stilbene) (0.1 mmol for each component), catalysts containing [Pt] (0.0025 mmol), THF (1.5 mL), PH_2: 1 bar, T: 30 ℃.

Supplementary Table 1. Composition of Pt-Ni nanostructures

Entry	Analyzed molar ratios (Pt-Ni) by ICP-MS	Analyzed molar ratios (Pt-Ni) by EDS
Pt-Ni Polyhedra	0.11 0.89	0.13 0.87
Pt-Ni Frame	0.68 0.32	0.71 0.29
Pt-Ni Frame @ MOF	0.13 0.87	0.16 0.84

Supplementary Table 2. Catalytic activity comparison among Ni based materials and Pt-Ni frame @ MOF

Entry	Conv. (%)
Pt-Ni frame@Ni-MOF-74 [a]	>99.0
Ni nanocrystal [a]	N.D.
Ni-MOF-74 [a]	N.D.
Pt-Ni frame@Ni-MOF-74 [b]	>99.0
Ni nanocrystal [b]	N.D.
Ni-MOF-74 [b]	N.D.
Pt-Ni frame@Ni-MOF-74 [c]	>99.0
Ni nanocrystal [c]	N.D.
Ni-MOF-74 [c]	N.D.

[a] 1-chloro-2-nitrobenzene (0.3 mmol), [Ni] (10 mol%), CH_3OH (1.5 mL), PH_2: 1 bar, T: 30 ℃, 3 h.

[b] styrene (0.1 mmol), [Ni] (10 mol%), THF (1.5 mL), PH_2: 1 bar, T: 30 ℃, 3 h.

[c] nitrobenzene (0.2 mmol), benzaldehyde (0.3 mmol), [Ni] (10 mol%), C_2H_5OH (2 mL), PH_2: 1 bar, T: 30 ℃, 3 h.

Supplementary Table 3. CO chemsorption measurements at 323K

CO chemsorption measurements at 323K

Entry	Sample	Metal(0) content （wt. %）	CO chemsorption (umol/g $_{catalyst}$)	active sites per gram metal * (umol/g $_{metal}$)	Dispersion** (mol $_{active\ sites}$/ mol $_{metal}$,%)
1	Pt-Ni frame	100	256.4	256.4	3.8
2	Ni-MOF-74	0	4.5	-	-
3	Pt-Ni frame @ MOF	19	35.5	159.5	2.4
4	Pt-Ni frame on MOF	19	45.5	209.5	3.1
5	Pt/C***	5	189.7	3794	73.8

The active site number and dispersion were calculated assuming a stoichiometry of one CO molecule per surface metal atom (metal atom = Pt + Ni).

*From entry1 and 2, it is showed that the CO chemsorption on Ni-MOF-74 is significantly lower than Pt-Ni frame core. In this column we deducted the chemsorption of Ni-MOF-74 part and normalize the CO uptake amount based on the total metal weight (Pt + Ni) of Pt-Ni nanostructures and Pt/C. We assume a stoichiometry of one CO molecule per surface active site to calculate the active sites per gram metal.

**As it is hard to exclude the catalytic role of Ni metal due to the possible synergetic interaction between Pt and Ni atoms, we calculate the dispersion of Pt-Ni nanostructures base on the total metal number (Pt + Ni).

***It has been inferred that carbon materials did not show CO chemsorption capability[2].

Supplementary References

1 Li, G. *et al.* Hydrogen storage in Pd nanocrystals covered with a metal–organic framework. *Nature Mater.* **2014**, *13*, 802–806.

2 Kirilin, A. V. *et al*. Aqueous-phase reforming of xylitol over Pt/C and Pt/TiC-CDC catalysts: catalyst characterization and catalytic performance. *Catal. Sci. Technol.* **2014**, 387–401.
